# China's top 10 breakthroughs in science and technology in 2024

**DOI:** 10.1093/nsr/nwaf187

**Published:** 2025-05-14

**Authors:** He Zhu

**Affiliations:** Science and news editor at the editorial office of *NSR* Beijing, China; Editorial office of *NSR* Beijing, China

## Abstract

For 2024, the Chinese Academy of Sciences and Chinese Academy of Engineering once again selected great achievements in a diverse array of fields including space and earth exploration, computer technology, bio-medicine and basic sciences.

**Figure:**
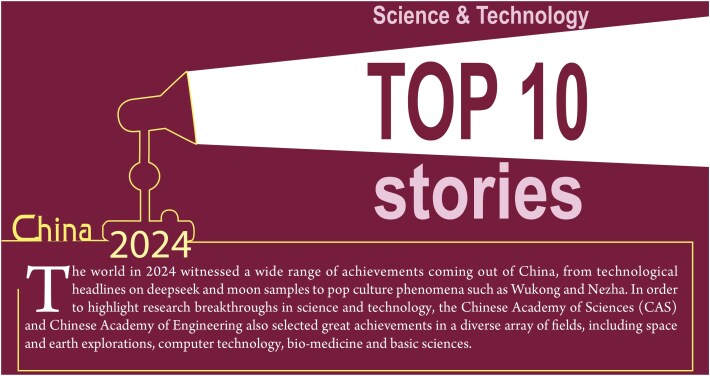


## CHANG'E-6 RETURNS SOIL SAMPLES FROM THE FAR SIDE OF THE MOON FOR THE FIRST TIME

Chang'e-6 completed its mission of collecting soil samples from the South Pole-Aitken (SPA) basin on the far side of the moon on 25 June, with 1935.3 grams of precious lunar regolith. Some basalt fragments in the samples were dated as 2.8 billion years old using isotope analysis. The origin of these fragments was interpreted to be volcanic activities in the SPA that were younger than those on the near side of the moon. Some other fragments in the sample indicated volcanic activities as old as 4.2 billion years and they consisted of rich concentrations of potassium, phosphorus and rare earth elements. This unprecedented achievement, first published on 15 November [1,2], advances our understanding of the origin and evolution of the moon.

**Figure 1.**
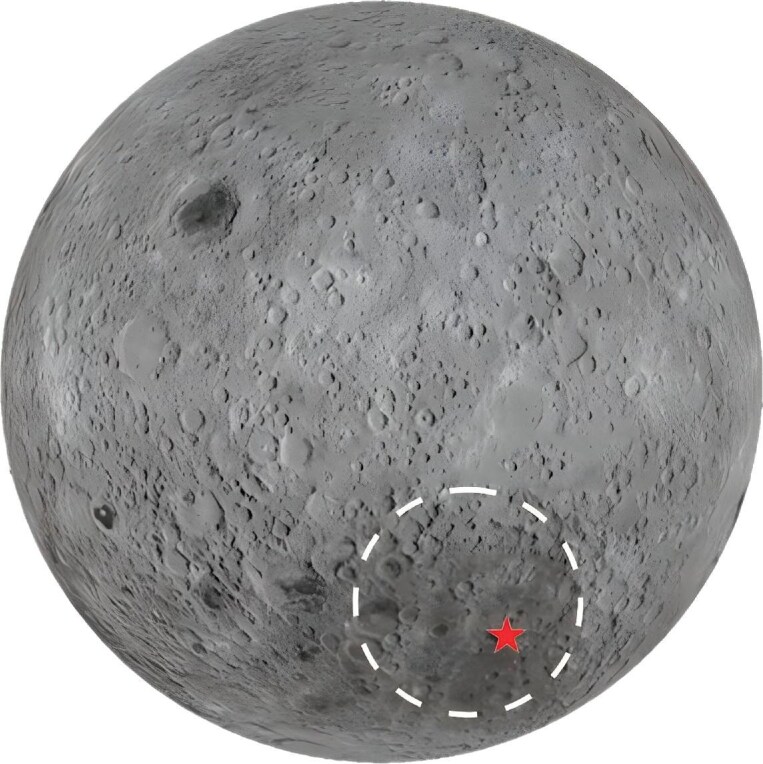
The landing site of Chang'e-6 is located in the South Pole-Aitken basin on the far side of the moon.

## CHINESE SCIENTISTS DEVELOPED THE FIRST PRIMITIVE-BASED VISION PROCESSOR WITH COMPLEMENTARY PATHWAYS

Researchers at the Center for Brain Inspired Computing Research (CBICR) at Tsinghua University developed a computer vision chip inspired by the human visual system [3]. This vision paradigm first parses visual information into primitive-based representations and then assembles these primitives to form two complementary pathways: one for accurate cognition and one for rapid response with fast processing. To test this paradigm in open-world applications, the research team developed a vision chip called ‘Tianmouc’ that achieves 10 000 frames per second and a dynamic range of 130 dB with its operation bandwidth reduced by 90% adaptively. The Tianmouc chip was integrated in an autonomous driving system that demonstrated accurate, fast and reliable perception. This primitive-based approach overcomes fundamental hurdles in computer vision in open-world applications.

**Figure 2.**
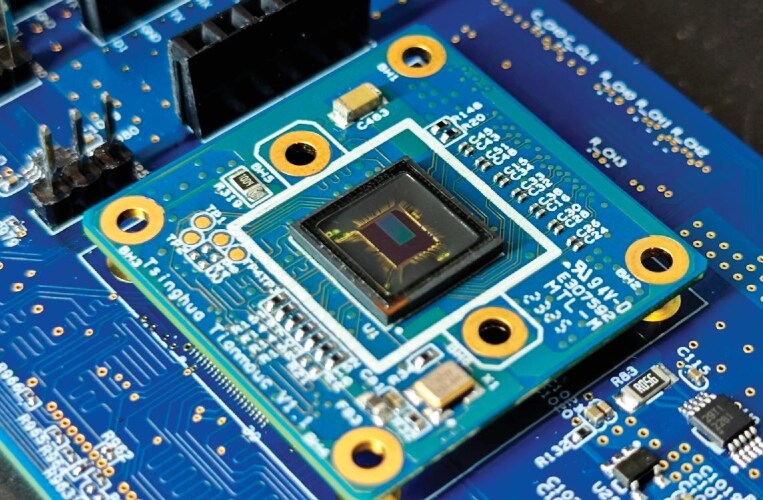
The vision chip ‘Tianmouc’ is first implemented in an autonomous driving system.

## CHINA'S FIRST OCEAN DRILL SHIP ‘MENG XIANG’ SETS SAIL

The first domestically designed and built drill ship ‘Meng Xiang’ launched on 11 November in Guangzhou. This ship was part of a major science and technology project in the 14th 5-year plan. With a maximal drill depth of 11 000 meters, its drilling capacity, research capacity and number of smart technologies all make it one of the best in the world, while its operational cost is the lowest in the world. The operational roles of ‘Meng Xiang’ include ocean drilling and oil and gas exploration, which are of strategic importance to the State. ‘Meng Xiang’ may be the first vessel in the world to realize the dream of ‘drilling through the crust and reaching the upper mantle’ to acquire deep earth resources, and it will advance China's plan to enter, survey and harvest the deep sea. ‘Meng Xiang’ was commissioned by the National Development and Reform commission and the Ministry of Natural Resources and managed by China Geological Survey. It was designed by the Marine Design and Research Institute of China and built by China State Shipbuilding Corporation Limited.

**Figure 3.**
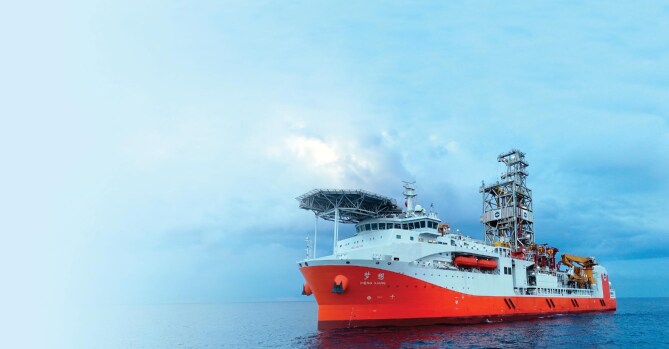
The ‘Meng Xiang’ ship is commissioned to enter, survey and harvest the deep sea.

## FIRST OPTICAL STORAGE DEVICE WITH PETABIT CAPACITY

Researchers at the Shanghai Institute of Optical and Fine Mechanics of CAS and University of Shanghai for Science and Technology made a breakthrough in 3D storage devices of super capacity and super resolution, gaining access to core technology in information storage [4]. Profs. Min Gu, Hao Ruan, and Jing Wen from these institutions, developed a photoresist film doped with aggregation-induced emission dye that can be excited by a femtosecond laser. The highly transparent property of the film expands its planar recording architecture to 3D, reaching hundreds of layers while its dual-beam method results in a recording spot enabling a super resolution. This technology reaches a storage capacity in the petabit range, with 54 nanometer recording spots and 100 layers, equivalent to 8000 existing optical disks or 100 hard drives. Its lifetime is expected to exceed 40 years. As current storage technologies such as semiconductor flash devices and hard disk drives have high energy requirements, this type of energy-efficient and high-capacity storage technology is essential to sustain and grow BigData tasks and large language models in this era of ever-growing demands on data storage.

**Figure 4.**
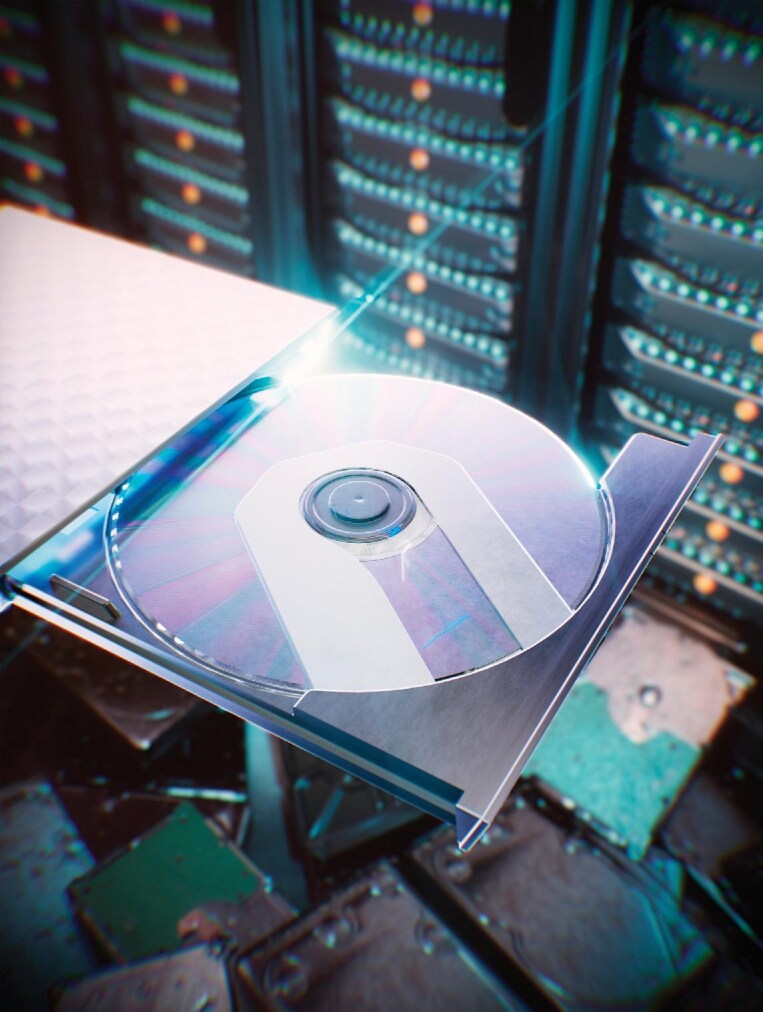
A highly transparent optical disk enables 3D storage, reaching petabit capacity.

## ‘TIANGUAN’ SATELLITE LAUNCHES AND RETURNS SURVEY DATA

‘TianGuan’ satellite was launched in Xichang Satellite Launch Center on 9 January 2024. The satellite, also called ‘Einstein Probe’, was designed, built and operated by various institutes in CAS in collaboration with the European Space Agency, Max Planck Institute for Extraterrestrial Physics and the French National Space Agency [5]. The mission control of TianGuan released the first batch of survey images on 27 April, initiating optical and radio surveys by multiple international teams. TianGuan reached its final orbit on 31 October and its control was handed over to the National Astronomical Observatories of CAS. The TianGuan satellite is regarded as a hunter of cosmic X-ray bursts. It can quickly characterize transients or outbursts using an onboard X-ray telescope. It will also monitor the changes of X-ray outbursts from white dwarfs, neutron stars and blackholes in our galaxy and neighboring galaxies. Furthermore, it has the potential to detect X-ray components from sources of neutrino or gravitational waves and provide insights into their formation, evolution, merger and other processes.

**Figure 5.**
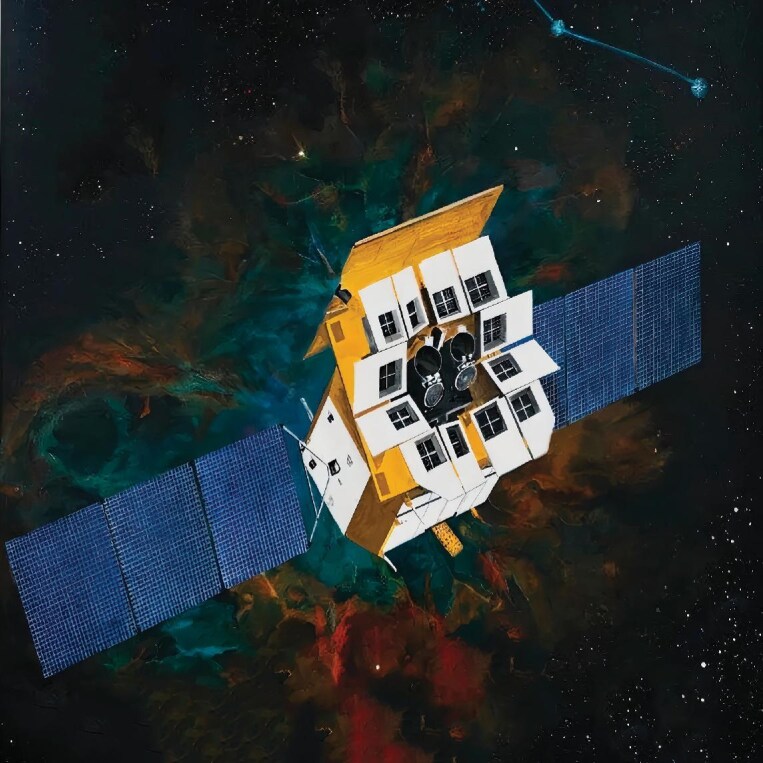
The ‘TianGuan’ satellite was developed by CAS with the participation of the Max Planck Institute, the French National Space Agency and the European Space Agency.

## A NEW APPROACH IN HELIUM-FREE CRYOGENIC TECHNOLOGY

Researchers from the Institute of Theoretical Physics (IOTP), Institute of Physics (IOP) and Beihang University discovered an exotic quantum state in condensed matter that combines the incompressibility of a solid and zero viscosity of a superfluid [6]. This supersolid state, called the spin supersolid, was realized in a cobalt-based antiferromagnet Na_2_BaCo(PO_4_) with a triangular lattice. In a demagnetization cooling process with the lowest temperature below 100 mK, researchers observed a giant magnetocaloric effect, i.e. changing a material's temperature by exposing it to a changing magnetic field. Evidence of this phase was also discovered in neutron diffraction experiments as the coexistence of three-sublattice spin solid order and interlayer incommensurability. The desirable properties of this phase of matter create promising possibilities in new devices in sub-kelvin refrigeration, especially when Helium-based refrigeration faces challenges due to its shortage. The lead researchers of this effort include Profs. Gang Su and Wei Li of IOTP, Profs. Peijie Sun and Junsen Xiang of IOP and Wentao Jin of Beihang University.

**Figure 6.**
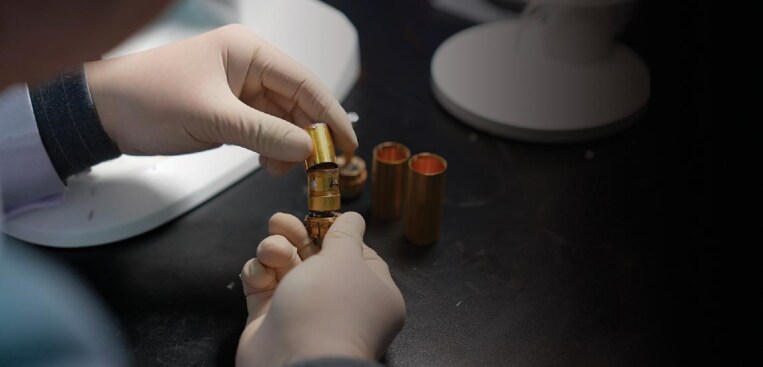
This new cryogenic technology reaches below 100 mK without using Helium.

## REFRACTORY AUTOIMMUNE DISEASES TREATED WITH GENETICALLY ENGINEERED CAR-T CELLS

Prof. Huji Xu of Naval Medical University in Shanghai, with colleagues from East China Normal University and BRL Medicine Inc., made a breakthrough in immunotherapy [7]. Using allogeneic chimeric antigen receptor (CAR-T) cells derived from healthy donors, they implemented genetic engineering with CRISPR-Cas9 to treat immune rejection of a patient with refractory immune-mediated necrotizing myopathy and two patients with diffuse cutaneous systemic sclerosis. The positive effects of this treatment were demonstrated by the significant improvement of index scores of clinical responses and the observed reversals of inflammation and fibrosis. This clinical study shows the safety and promise of CAR-T cells in treating severe refractory autoimmune diseases. The expansion of this type of treatment is underway to bring hope to more autoimmune patients.

**Figure 7.**
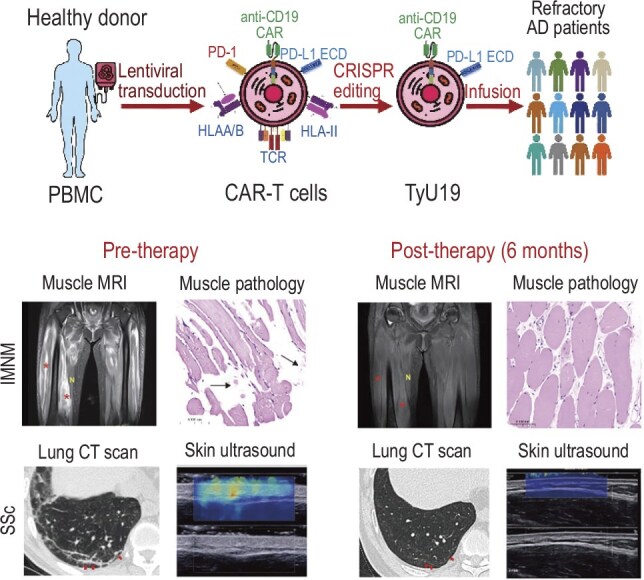
CAR-T therapy demonstrates significant improvement after treatment.

## MESOSCOPIC 3D IMAGING OF ORGANS TO RESOLVE LARGE-SCALE INTERCELLULAR DYNAMICS

Non-invasive imaging of structures and functions of organs, particularly the interactions of cells within, are imperative in understanding organs’ physio-pathological processes. Technical issues stemming from higher spatial and temporal resolutions, optical heterogeneity, and phototoxicity all present challenges along the path to reaching the mesoscopic scale. Prof. Qionghai Dai of Tsinghua University led his research team in designing a compact real-time, ultra-large-scale, high-resolution 3D mesoscope (RUSH3D), with a spatial resolution of 2.6 × 2.6 × 6 μm^3^ in a volume of 8000 × 6000 × 400 μm^3^ and a temporal resolution of 20 Hz with low phototoxicity [8]. Compared to previous technologies, RUSH3D achieves one order-of-magnitude improvement in data throughput and one order-of-magnitude reduction in size and cost. The advancement of imaging capability in the new system includes heterogeneous immune responses following traumatic brain injury at single-cell resolution and premovement neural activity of the mouse cortex. The development and commercialization of RUSH3D positions Chinese researchers at the frontier of *in vivo* mesoscopic microscopy. Various research institutions in China currently rely on RUSH3D for innovative experiments in oncology, immunology and neuroscience.

**Figure 8.**
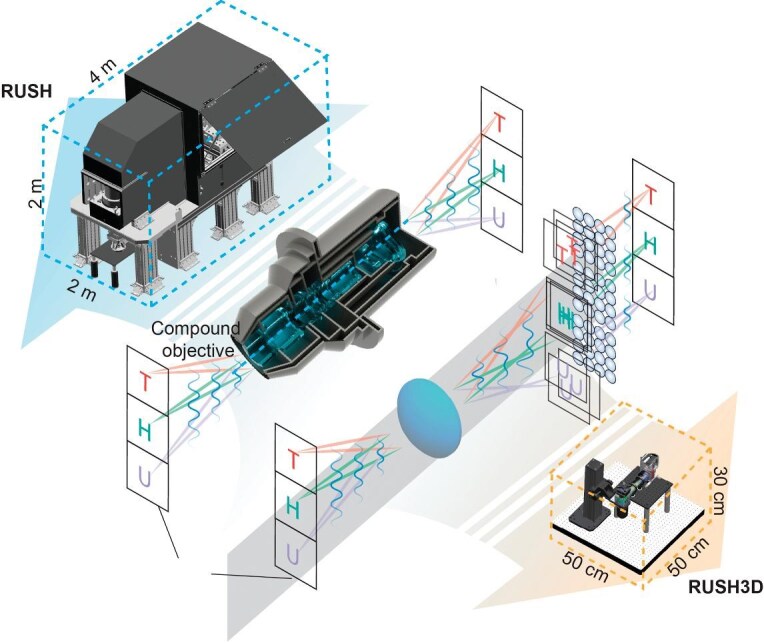
RUSH3D excels both in imaging range and resolution.

## CHIRAL GRAVITON MODES DISCOVERED IN FRACTIONAL QUANTUM HALL LIQUIDS

Chiral graviton modes (CGMs) are proposed as quanta of fluctuations of an internal quantum metric in a condensed-matter system. CGMs are considered to be analogous to gravitons, hypothetical particles that underlie gravity of matter. However, much like gravitons, real-world observation of CGM remains a challenge. Prof. Lingjie Du of Nanjing University along with collaborators at Columbia University, Princeton University and the University of Munster, detected CGM in fractional quantum Hall (FQH) liquids in GaAs quantum wells. The research team used a self-designed and assembled device called a low-temperature resonant inelastic scattering system with circularly polarized light [9]. This discovery marks the first-in-the-world observation of a quasi-particle with characteristics of gravitons. Although a CGM is not an elemental particle such as a graviton, its discovery in a condensed-matter system still provides evidence for a new geometric description of FQH and may bring forth new physics of quantum metric effects in topological correlated systems.

**Figure 9.**
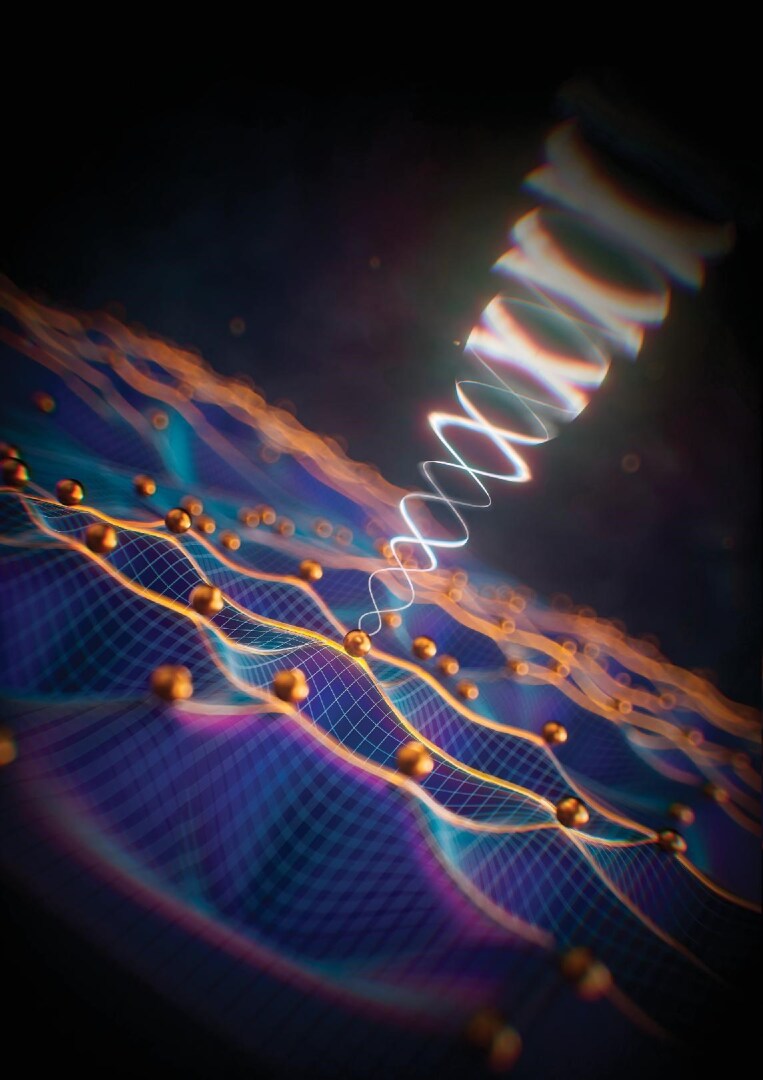
This quasi-particle in a condensed-matter system shows properties similar to those of gravitons.

## THE WORLD'S LONGEST MOUNTAIN ICE CORE ACQUIRED IN THE SECOND QINGHAI-TIBET EXPEDITION

China initiated, on 18 August, the second Qinghai-Tibet expedition themed ‘Guarding the reservoir: one glacier, two lakes and three rivers’. This expedition focused on the largest glacier in low-to-mid latitude regions of the world, the Purog Kangri ice sheet, the two largest lakes in Tibet, Siling Co and Nam Co, and three rivers originating from the Qinghai-Tibet region: the Yangtze, the Nujiang and the Yarlung Zangbo. Led by 10 academicians of CAS, the expedition team discovered the thickest glacier in Purog Kangri ice sheet and set the world's drilling record of the longest mountain ice core at 324 meters. Several first-ever achievements were accomplished during this expedition. They include observation of the climate transition process from Monsoon to Westerly using tethered aerostats; identification of 2 suspected new species and more than 20 regionally new recorded species in the ice sheet region; discovery of a concentrated rare metal beryllium near the main peak of the Nyenchen Tanglha Mountains; and drilling of over 1000 meters in the Lunpola Basin.

**Figure 10.**
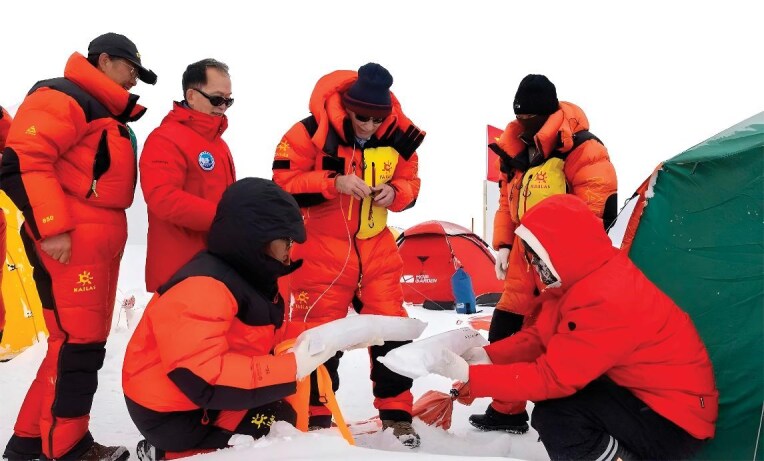
The second Qinghai-Tibet expedition sets a new world record for the longest mountain ice core at 324 meters.

## Author Biography

He Zhu (朱禾) is a science and news editor at the editorial office of *NSR* in Beijing, China. This article's art design was carried out by Xiaoling Yu (于晓玲) at the editorial office of *NSR* in Beijing, China.
